# A case-control study of osteopathic palpatory findings in type 2 diabetes mellitus

**DOI:** 10.1186/1750-4732-1-6

**Published:** 2007-02-08

**Authors:** John C Licciardone, Kimberly G Fulda, Scott T Stoll, Russell G Gamber, A Clifton Cage

**Affiliations:** 1 Osteopathic Research Center, University of North Texas Health Science Center-Texas College of Osteopathic Medicine, Fort Worth, TX 76107, USA; 2 Department of Family Medicine, University of North Texas Health Science Center-Texas College of Osteopathic Medicine, Fort Worth, TX 76107, USA

## Abstract

**Background:**

Although type 2 diabetes mellitus is often managed by osteopathic physicians, osteopathic palpatory findings in this disease have not been adequately studied.

**Methods:**

A case-control study was used to measure the association between type 2 diabetes mellitus and a series of 30 osteopathic palpatory findings. The latter included skin changes, trophic changes, tissue changes, tenderness, and immobility at spinal segmental levels T5–T7, T8–T10, and T11-L2 bilaterally. Logistic regression models that adjusted for age, sex, and comorbid conditions were used to compute odds ratios (ORs) and 95% confidence intervals (CIs) for the associations between type 2 diabetes mellitus and each of these findings.

**Results and discussion:**

A total of 92 subjects were included in the study. After controlling for age, sex, hypertension, and clinical depression, the only significant finding was an association between type 2 diabetes mellitus and tissue changes at T11-L2 on the right side (OR, 5.54; 95% CI, 1.76–17.47; P = .003). Subgroup analyses of subjects with type 2 diabetes mellitus and hypertension demonstrated significant associations with tissue changes at T11-L2 bilaterally (OR, 27.38; 95% CI, 1.75–428; P = .02 for the left side and OR, 24.00; 95% CI, 1.51–382; P = .02 for the right side). Among subjects with type 2 diabetes mellitus and hypertension, there was also a strong diabetes mellitus duration effect for tissue changes at T11-L2 bilaterally (OR, 12.00; 95% CI, 1.02–141; P = .05 for short duration vs. OR, 32.00; 95% CI, 2.29–448; P = .01 for long duration on the left side; and OR, 17.33; 95% CI, 1.39–217; P = .03 for short duration vs. OR, 32.00; 95% CI, 2.29–448; P = .01 for long duration on the right side).

**Conclusion:**

The only consistent finding in this study was an association between type 2 diabetes mellitus and tissue changes at T11-L2 on the right side. Potential explanations for this finding include reflex viscerosomatic changes directly related to the progression of type 2 diabetes mellitus, a spurious association attributable to confounding visceral diseases, or a chance observation unrelated to type 2 diabetes mellitus. Larger prospective studies are needed to better study osteopathic palpatory findings in type 2 diabetes mellitus.

## Background

Type 2 diabetes mellitus is an important public health problem that comprises 90% to 95% of the estimated 18.2 million Americans with diabetes mellitus [1]. Although type 2 diabetes mellitus was previously called non-insulin-dependent diabetes mellitus or adult-onset diabetes mellitus, neither term adequately describes the disease. Type 2 diabetes mellitus usually begins as insulin resistance and, as the need for insulin rises, the pancreas may lose its ability to produce insulin, thereby requiring exogenous insulin. The prevalence of type 2 diabetes mellitus in children and adolescents is increasing as a consequence of the continuing rise in obesity in this population [2]. The cost of diabetes mellitus was estimated to be $132 billion in the United States during 2002, including both medical expenditures and lost productivity [3].

Prediabetes is a term used to identify persons at high risk of developing diabetes mellitus because they have elevated fasting glucose or impaired glucose tolerance findings that are not sufficiently abnormal to meet the criteria for diagnosis of diabetes mellitus. About 41 million Americans aged 40–74 years are estimated to have prediabetes [1]. Identifying and intervening in persons with prediabetes may help prevent or delay the progression to diabetes mellitus and its complications, including blindness, kidney damage, and lower-limb amputations.

Research that addresses osteopathic palpatory findings or osteopathic manipulative treatment (OMT) in patients with diabetes mellitus is scarce. The spinal segmental sites for somatic dysfunction associated with visceral diseases, such as diabetes mellitus, are thought to be related to the autonomic nervous system supply for the affected organ [4]. For example, segmental sympathetic nerve supply to the pancreas is generally localized to T5–T11 [4]. Using a poorly described methodology over a 25-year period, a series of 150 diabetic and non-diabetic patients received OMT that was intended to provide "pancreatic stimulation" [5]. Baseline fasting blood glucose measures were recorded prior to receiving OMT and were repeated 30 or 60 minutes (or both) after receiving OMT. A rapid drop in blood glucose following OMT was reported, including one case of hypoglycemic coma. In a complementary phase of this study [5], 40 apparently non-diabetic patients received OMT that was intended to provide "pancreatic inhibition." Baseline fasting blood glucose measures were recorded prior to receiving OMT and both 30 and 60 minutes after receiving OMT. A rapid increase in blood glucose was reported in this phase of the study. Results such as those described above have not been replicated and reported in the contemporary osteopathic literature.

In theory, OMT for the somatic manifestations of visceral disease may not only improve the affected paraspinal tissues, but may also effect a disruption of the viscerosomatic reflex arc, thereby creating the potential for amelioration of the underlying visceral disease [4]. Nevertheless, clinical studies to support this theoretical concept are lacking. The specification of osteopathic palpatory findings associated with a particular chronic disease has heretofore been an important barrier in performing such studies. Thus, we conducted a case-control study to identify the osteopathic palpatory findings associated with type 2 diabetes mellitus.

## Methods

### Study design

A case-control study was used to measure the association between type 2 diabetes mellitus and various osteopathic palpatory findings. There is a temporal lag between the pathogenesis and progression of type 2 diabetes mellitus and any subsequent osteopathic palpatory finding that potentially may be detected. Therefore, the presence or absence of type 2 diabetes mellitus was the independent (exposure) variable and the observed osteopathic palpatory findings were the dependent ("disease") variables in this study. Thus, subjects were labelled as cases or controls, respectively, if a given osteopathic palpatory finding was present or absent. In this manner, a series of case-control analyses were performed – one for each of the 30 osteopathic palpatory findings described below. Subjects were recruited for this study and enrolled between April 2002 and November 2003 at the University of North Texas Health Science Center-Texas College of Osteopathic Medicine (UNTHSC-TCOM). Subjects were primarily recruited from patients attending clinics affiliated with the Department of Family Medicine, although some subjects with type 2 diabetes mellitus were also recruited from other university clinics and from the Fort Worth metropolitan area. The Institutional Review Board at UNTHSC approved all research procedures.

Subject eligibility was determined using a standard screening procedure for university-based clinic patients. Patients were excluded from further participation if they met any of the following criteria: (1) age < 18 years or age >69 years; (2) diagnosis of type 1 diabetes mellitus; (3) history of any of the following pancreatic or related disorders: (a) acute or chronic pancreatitis, (b) pancreatic tumor or cancer, (c) Zollinger-Ellison syndrome, or (d) any other medical or surgical condition resulting in functional hypo- or hyperglycemia; (4) history of surgery involving the pancreas; (5) current pregnancy; (6) history of pregnancy within the last three months or intent to become pregnant within the next six months; (7) history of back surgery within the last three months; (8) potential contraindications to receiving OMT, including: (a) cancer other than non-malignant skin cancer, (b) torn tissue or hemorrhage in the back, (c) spinal osteomyelitis, (d) spinal fracture, (e) herniated disc, (f) ankylosing spondylitis, or (g) cauda equina syndrome; or (9) receipt of Workers' Compensation benefits within the last three months. Several of these exclusion criteria were primarily intended for the follow-up study not reported herein. Identical exclusion criteria were used for non-university-based clinic patients. All subjects were required to provide informed consent prior to participating in the study.

### Type 2 diabetes mellitus status

Institutional medical records were used to confirm a clinical diagnosis of type 2 diabetes mellitus for university-based clinic subjects. Structured face-to-face interviews by trained study personnel were used to confirm a diagnosis of type 2 diabetes mellitus for non-university-based clinic subjects. During these interviews, a comprehensive medical history was acquired, including the history of type 2 diabetes mellitus onset, progression, and current management. The absence of type 2 diabetes mellitus in subjects was confirmed by institutional medical records that did not support a clinical or laboratory diagnosis of type 2 diabetes mellitus within the last year.

### Osteopathic palpatory examination

Each subject was independently examined by two osteopathic manipulative medicine fellows during the same clinic visit to determine if osteopathic palpatory findings were present or absent. The fellows in this study were select medical students who elected to undertake an additional year of osteopathic manipulative medicine training during their undergraduate medical curriculum. Overall, six fellows performed these examinations during their clinical rotations dedicated to fellowship training. Prior to participation, fellows received training in the research protocol provided by senior osteopathic manipulative medicine faculty. This training included standard criteria for determining the presence or absence of osteopathic palpatory findings [6].

Fellows were blinded to the type 2 diabetes mellitus status of subjects during the examination. Subjects were instructed not to discuss any health-related matters during the examination. Three fellow pairs were used throughout the study to perform all osteopathic palpatory examinations. In the case of discrepant osteopathic palpatory findings between fellows who examined the same subject, the findings were randomly recorded as either the first or second fellow's set of observations for that subject.

Somatic dysfunction is defined as impaired or altered function of related components of the somatic (body framework) system: skeletal, arthrodial, and myofascial structures, and related vascular, lymphatic, and neural elements [6]. The osteopathic palpatory examination in this study assessed five elements that are manifestations of somatic dysfunction potentially associated with a chronic disease such as type 2 diabetes mellitus. These elements, which often are thought to be secondary to chronic sympathetic activity, included the following [7]: (1) skin changes (coolness or paleness); (2) trophic changes (dry or scaly skin, pimples, folliculitis, or abnormal pigmentation); (3) tissue changes (doughy, ropy, thickened, or fibrotic interstitial tissues); (4) tenderness; and (5) immobility (restricted motion). These examinations included spinal segmental levels T5–T7, T8–T10, and T11-L2 bilaterally. Thus, the presence or absence of 30 osteopathic palpatory findings (five elements of somatic dysfunction × three ranges of spinal segmental levels × two sides) was determined during these examinations.

### Interexaminer reliability

The interexaminer reliability for each of the 30 osteopathic palpatory findings was initially measured by the crude proportional agreement between the fellows who each examined the same subjects. Thus, this measure could potentially range from 0 (no agreement at all) to 1 (perfect agreement). Subsequently, Cohen's kappa was used to measure the interexaminer agreement between fellows for each element of somatic dysfunction after adjusting for chance agreement [8]. As with proportional agreement, a kappa value of 1 indicates perfect agreement between examiners. A kappa value of 0 indicates a level of agreement between examiners that would be expected to occur by chance alone. Unlike proportional agreement, however, kappa can take on negative values if the observed level of agreement between examiners is less than that expected by chance alone. Interexaminer reliability was also measured for osteopathic palpatory findings aggregated according to element of somatic dysfunction, spinal segmental level, and laterality.

### Data management and analysis

Initially, we planned to include subjects with and without type 2 diabetes mellitus who were matched according to age, sex, and race/ethnicity. However, during the course of the study, it became apparent that less than half of the subjects with type 2 diabetes mellitus would have suitable matches without type 2 diabetes mellitus. To avoid a substantial loss of statistical power, we subsequently included all subjects, regardless of matching, and used logistic regression to control for potential confounders. Hence, there were substantially more subjects with than without type 2 diabetes mellitus.

Logistic regression was used to compute the crude odds ratios (ORs) and 95% confidence intervals (CIs) for the associations between type 2 diabetes mellitus and each of the 30 osteopathic palpatory findings. Age- and sex-adjusted ORs and 95% CIs were then computed using partially-adjusted logistic regression models. Finally, fully-adjusted logistic regression models were used, which also included commonly observed comorbid conditions as covariates. Data analyses were performed with the Systat (Systat Software, Inc, Point Richmond, CA, USA) and Excel (Microsoft Corporation, Redmond, WA, USA) software packages. Hypotheses were tested using two-tailed assumptions at the .05 level of statistical significance.

## Results

### Subject characteristics

There were 60 subjects with type 2 diabetes mellitus and 32 subjects without type 2 diabetes mellitus included in the study. The characteristics of these subjects are presented in Table 1. There were no significant differences between subjects with and without type 2 diabetes mellitus with respect to sociodemographic characteristics; however, subjects with type 2 diabetes mellitus were more likely to have hypertension (P < .001) and clinical depression (P = .05) than subjects without type 2 diabetes mellitus. Other comorbid conditions occurred too infrequently to perform meaningful statistical analyses. The median duration of type 2 diabetes mellitus since diagnosis was five years (range, 0.1 to 25 years).

**Table 1:** Characteristics of subjects with and without type 2 diabetes mellitus.*

	**Diagnosis of Type 2 Diabetes Mellitus**		
		
	**Yes**	**No**	
		
**Subject Characteristic**	**No. (%)**	**No. (%)**	**P**
			
Age, yr†	48.8 (10.0)	49.6 (12.3)	.73
			
Sex			.25
Female	30 (50)	20 (63)	
Male	30 (50)	12 (38)	
			
Race/ethnicity			.43
White	36 (60)	23 (72)	
Black	20 (33)	8 (25)	
Hispanic	3 (5)	0 (0)	
Asian/Pacific Islander	1 (2)	1 (3)	
			
Marital status			.93
Single	18 (30)	10 (31)	
Married	23 (38)	14 (44)	
Separated	4 (7)	1 (3)	
Divorced	12 (20)	6 (19)	
Widowed	3 (5)	1 (3)	
			
Comorbid conditions			
Hypertension			< .001
Yes	34 (57)	5 (16)	
No	26 (43)	27 (84)	
Clinical depression			.05
Yes	23 (38)	6 (19)	
No	37 (62)	26 (81)	

*Percentages may not sum to 100 because of rounding.

†Table entries are mean (SD).

### Interexaminer reliability

**Figure 1:**
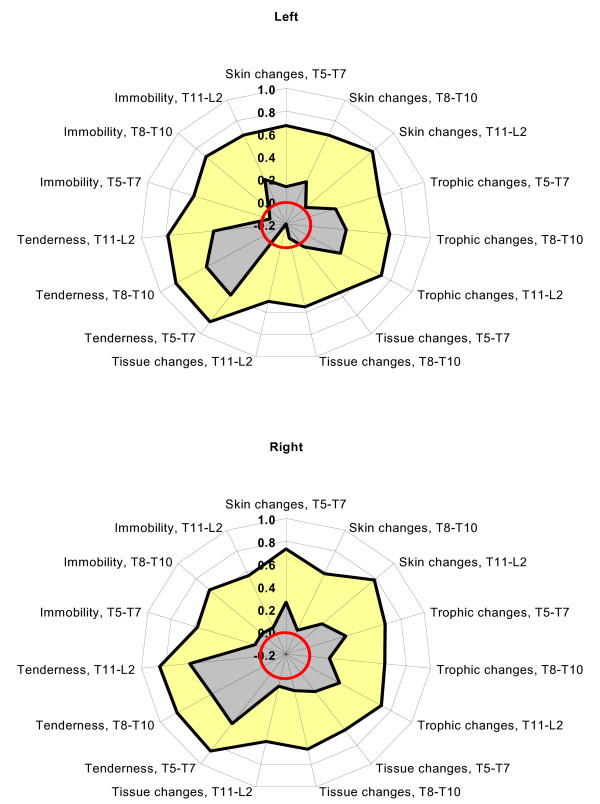
Radar plots of proportional agreement and kappa for osteopathic palpatory findings according to element of somatic dysfunction, spinal segmental level, and laterality. The proportional agreement is presented in yellow and kappa in gray. The red circle represents zero on each radial scale.

The overall proportional agreement (and kappa) for each of the five elements of somatic dysfunction was: 0.69 (0.16) for skin changes; 0.66 (0.28) for trophic changes; 0.58 (0.05) for tissue changes; 0.84 (0.54) for tenderness; and 0.62 (0.09) for immobility. The levels of interexaminer reliability did not vary much according to spinal segmental level: 0.67 (0.34) for T5–T7, 0.67 (0.35) for T8–T10, and 0.69 (0.36) for T11-L2. Similarly, there was little variation in interexaminer reliability according to laterality: 0.67 (0.34) for left-, and 0.68 (0.36) for right-sided elements of somatic dysfunction. When all palpatory findings were aggregated, the interexaminer reliability was 0.68 (0.35). The interexaminer reliability of osteopathic palpatory findings according to element of somatic dysfunction, spinal segmental level, and laterality is presented in Table 2. Figure 1 presents the corresponding radar plots that illustrate the relationship between proportional agreement and kappa.

**Table 2:** Interexaminer reliability of osteopathic palpatory findings according to element of somatic dysfunction, spinal segmental level, and laterality.*

**Element of Somatic Dysfunction**	**Spinal Segmental Level**	**Laterality**	
		
		**Left**	**Right**
			
Skin changes			
	T5–T7	0.67 (0.13)	0.73 (0.26)
	T8–T10	0.66 (0.21)	0.58 (0.03)
	T11-L2	0.76 (0.02)	0.78 (0.20)
			
Trophic changes			
	T5–T7	0.61 (0.23)	0.66 (0.32)
	T8–T10	0.66 (0.30)	0.62 (0.16)
	T11-L2	0.71 (0.32)	0.71 (0.31)
			
Tissue changes			
	T5–T7	0.55 (0.05)	0.63 (0.21)
	T8–T10	0.55 (-0.07)	0.66 (0.13)
	T11-L2	0.50 (-0.20)	0.59 (0.09)
			
Tenderness			
	T5–T7	0.87 (0.58)	0.86 (0.56)
	T8–T10	0.85 (0.56)	0.84 (0.51)
	T11-L2	0.78 (0.40)	0.85 (0.60)
			
Immobility			
	T5–T7	0.60 (-0.06)	0.57 (0.07)
	T8–T10	0.69 (-0.01)	0.65 (0.07)
	T11-L2	0.66 (0.23)	0.56 (0.06)

*Table entries are for proportional agreement (and kappa).

### Prevalence of osteopathic palpatory findings

**Figure 2:**
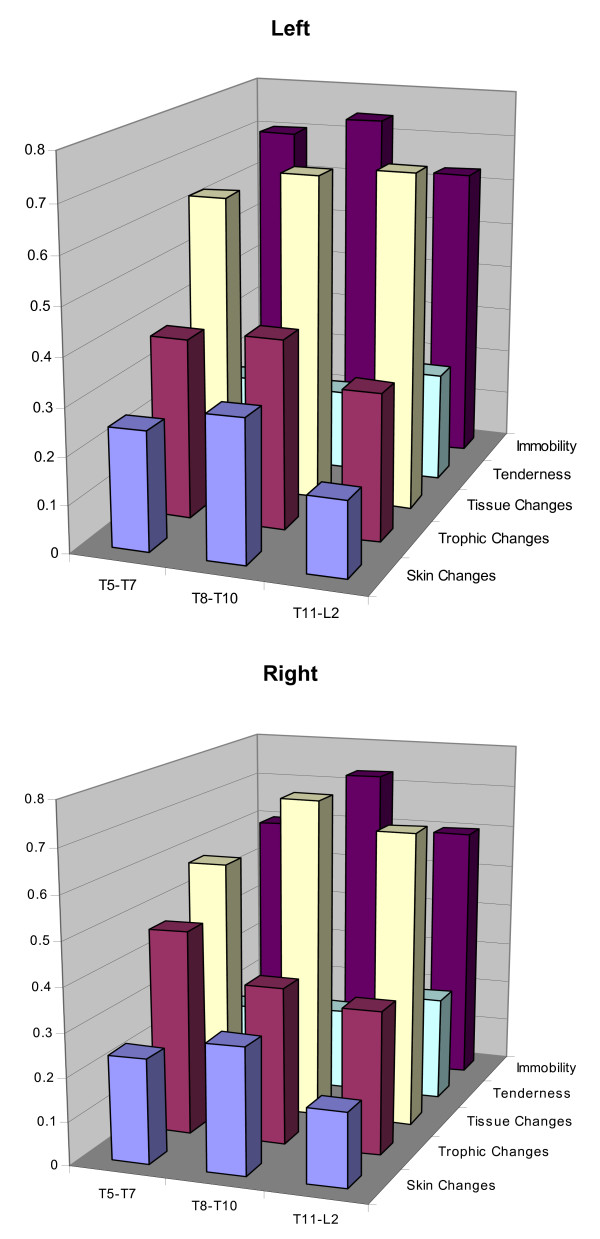
Columnar graphs of osteopathic palpatory findings according to element of somatic dysfunction, spinal segmental level, and laterality. The columns represent the proportion of subjects with a positive osteopathic palpatory finding.

The number of subjects with and without positive osteopathic palpatory findings according to element of somatic dysfunction, spinal segmental level, and laterality is presented in Table 3. The prevalence of these osteopathic palpatory findings (i.e., the proportion of subjects with positive osteopathic palpatory findings) is depicted in Figure 2. Immobility and tissue changes were the most common osteopathic palpatory findings.

**Table 3:** Osteopathic palpatory findings according to element of somatic dysfunction, spinal segmental level, and laterality.*

**Element of Somatic Dysfunction**	**Spinal Segmental Level**	**Laterality**	
		
		**Left**	**Right**
			
Skin changes			
	T5–T7	23/69	22/70
	T8–T10	27/63	27/65
	T11-L2	15/76	16/76
			
Trophic changes			
	T5–T7	34/56	42/48
	T8–T10	36/55	33/58
	T11-L2	28/63	30/62
			
Tissue changes			
	T5–T7	57/34	53/38
	T8–T10	63/28	67/24
	T11-L2	64/26	62/29
			
Tenderness			
	T5–T7	17/75	17/75
	T8–T10	15/74	17/73
	T11-L2	21/70	22/70
			
Immobility			
	T5–T7	64/27	54/36
	T8–T10	67/24	67/25
	T11-L2	58/34	55/36

*Table entries are for subjects with/without a positive osteopathic palpatory finding. These subjects represent cases/controls in the study.

### Associations between type 2 diabetes mellitus and osteopathic palpatory findings

The osteopathic palpatory findings according to element of somatic dysfunction, spinal segmental level, and laterality are presented along with the corresponding crude ORs and 95% CIs in Table 4. Type 2 diabetes mellitus was significantly associated with three osteopathic palpatory findings: tissue changes at T11-L2 on the right side (OR, 4.44; 95% CI, 1.73–11.37; P = .002); tenderness at T11-L2 on the left side (OR, 4.00; 95% CI, 1.08–14.86; P = .04); and immobility at T5–T7 on the right side (OR, 2.56; 95% CI, 1.05–6.25; P = .04).

**Table 4:** Crude odds ratios (ORs) and 95% confidence intervals (CIs) for the associations between type 2 diabetes mellitus (T2DM) and osteopathic palpatory findings according to element of somatic dysfunction, spinal segmental level, and laterality.*

		**Laterality**					
		
**Element of Somatic Dysfunction**	**Spinal Segmental Level**	**Left**			**Right**		
		
		**T2DM+**	**T2DM-**	**OR (95% CI)**	**T2DM+**	**T2DM-**	**OR (95% CI)**
							
Skin changes							
	T5–T7	16/44	7/25	1.30 (0.47–3.58)	15/45	7/25	1.19 (0.43–3.31)
	T8–T10	21/38	6/25	2.30 (0.82–6.50)	16/44	11/21	0.69 (0.27–1.75)
	T11-L2	12/48	3/28	2.33 (0.61–8.99)	12/48	4/28	1.75 (0.51–5.95)
							
Trophic changes							
	T5–T7	24/35	10/21	1.44 (0.58–3.59)	27/31	15/17	0.99 (0.42–2.34)
	T8–T10	25/34	11/21	1.40 (0.57–3.43)	24/35	9/23	1.75 (0.69–4.44)
	T11-L2	19/40	9/23	1.21 (0.47–3.12)	22/38	8/24	1.74 (0.67–4.52)
							
Tissue changes							
	T5–T7	38/21	19/13	1.24 (0.51–3.00)	36/23	17/15	1.38 (0.58–3.29)
	T8–T10	42/17	21/11	1.29 (0.51–3.25)	47/12	20/12	2.35 (0.90–6.11)†
	T11-L2	45/14	19/12	2.03 (0.79–5.19)	47/12	15/17	4.44 (1.73–11.37)‡
							
Tenderness							
	T5–T7	13/47	4/28	1.94 (0.57–6.52)	13/47	4/28	1.94 (0.57–6.52)
	T8–T10	11/46	4/28	1.67 (0.49–5.77)	14/44	3/29	3.08 (0.81–11.65)
	T11-L2	18/42	3/28	4.00 (1.08–14.86)§	17/43	5/27	2.13 (0.71–6.46)
							
Immobility							
	T5–T7	40/19	24/8	0.70 (0.27–1.85)	40/19	14/17	2.56 (1.05–6.25)§
	T8–T10	45/14	22/10	1.46 (0.56–3.81)	45/15	22/10	1.36 (0.53–3.52)
	T11-L2	39/21	19/13	1.27 (0.53–3.07)	34/25	21/11	0.71 (0.29–1.74)

*Table entries in the T2DM+ and T2DM- columns are the number of subjects with/without a positive osteopathic palpatory finding. These subjects represent cases/controls in the study. The ORs and 95% CIs are for risk of a positive osteopathic palpatory finding in subjects with T2DM vs. subjects without T2DM.

†Statistical trend, .05 ≤ P < .10

‡P = .002.

§P = .04.

As shown in Table 5, the three significant associations between type 2 diabetes mellitus and osteopathic palpatory findings persisted in the partially-adjusted logistic regression models that controlled for age and sex: tissue changes at T11-L2 on the right side (OR, 4.49; 95% CI, 1.69–11.96; P = .003); tenderness at T11-L2 on the left side (OR, 4.28; 95% CI, 1.13–16.20; P = .03); and immobility at T5–T7 on the right side (OR, 2.71; 95% CI, 1.09–6.75; P = .03).

**Table 5:** Partially-adjusted odds ratios (ORs) and 95% confidence intervals (CIs) for the associations between type 2 diabetes mellitus and osteopathic palpatory findings according to element of somatic dysfunction, spinal segmental level, and laterality.*

		**Laterality**	
		
**Element of Somatic Dysfunction**	**Spinal Segmental Level**	**Left**	**Right**
		
		**OR (95% CI)**	**OR (95% CI)**
			
Skin changes			
	T5–T7	1.18 (0.41–3.39)	0.99 (0.33–2.93)
	T8–T10	2.31 (0.81–6.61)	0.68 (0.26–1.77)
	T11-L2	2.12 (0.53–8.50)	1.81 (0.50–6.60)
			
Trophic changes			
	T5–T7	1.74 (0.65–4.67)	1.01 (0.42–2.42)
	T8–T10	1.34 (0.52–3.43)	1.78 (0.68–4.65)
	T11-L2	1.01 (0.36–2.80)	1.55 (0.57–4.21)
			
Tissue changes			
	T5–T7	1.22 (0.50–2.99)	1.36 (0.56–3.30)
	T8–T10	1.55 (0.59–4.06)	2.46 (0.93–6.53)†
	T11-L2	2.07 (0.80–5.39)	4.49 (1.69–11.96)‡
			
Tenderness			
	T5–T7	2.22 (0.64–7.68)	2.36 (0.67–8.27)
	T8–T10	2.01 (0.56–7.23)	3.56 (0.91–13.88)†
	T11-L2	4.28 (1.13–16.20)§	2.39 (0.77–7.46)
			
Immobility			
	T5–T7	0.69 (0.25–1.84)	2.71 (1.09–6.75)§
	T8–T10	1.44 (0.55–3.80)	1.45 (0.55–3.83)
	T11-L2	1.37 (0.55–3.41)	0.79 (0.32–1.96)

*Table entries are for risk of a positive osteopathic palpatory finding in subjects with type 2 diabetes mellitus vs. subjects without type 2 diabetes mellitus, adjusted for age and sex.

†Statistical trend, .05 ≤ P < .10.

‡P = .003.

§P = .03.

The fully-adjusted logistic regression models that controlled for age, sex, hypertension, and clinical depression are presented in Table 6. The only significant result in these analyses was an association between type 2 diabetes mellitus and tissue changes at T11-L2 on the right side (OR, 5.54; 95% CI, 1.76–17.47; P = .003). We subsequently performed a series of *post-hoc *subgroup analyses to explore whether tissue changes at the T11-L2 segmental level may be manifestations of a viscerosomatic reflex involving the kidney because its segmental sympathetic nerve supply closely corresponds to T11-L2 [4] and it is commonly affected in the progression of type 2 diabetes mellitus.

**Table 6:** Fully-adjusted odds ratios (ORs) and 95% confidence intervals (CIs) for the associations between type 2 diabetes mellitus and osteopathic palpatory findings according to element of somatic dysfunction, spinal segmental level, and laterality.*

		**Laterality**	
		
**Element of Somatic Dysfunction**	**Spinal Segmental Level**	**Left**	**Right**
		
		**OR (95% CI)**	**OR (95% CI)**
			
Skin changes			
	T5–T7	0.98 (0.29–3.24)	1.12 (0.32–3.86)
	T8–T10	1.88 (0.57–6.13)	0.54 (0.18–1.65)
	T11-L2	1.08 (0.22–5.29)	1.08 (0.24–4.79)
			
Trophic changes			
	T5–T7	0.83 (0.26–2.66)	0.43 (0.14–1.30)
	T8–T10	0.80 (0.27–2.40)	1.27 (0.42–3.84)
	T11-L2	0.31 (0.08–1.18)	0.82 (0.25–2.64)
			
Tissue changes			
	T5–T7	1.01 (0.37–2.74)	1.26 (0.47–3.39)
	T8–T10	1.33 (0.45–3.88)	1.95 (0.66–5.79)
	T11-L2	2.24 (0.76–6.60)	5.54 (1.76–17.47)†
			
Tenderness			
	T5–T7	1.48 (0.37–6.01)	1.60 (0.40–6.45)
	T8–T10	1.42 (0.34–5.94)	2.13 (0.49–9.34)
	T11-L2	3.60 (0.87–14.95)	2.52 (0.72–8.82)
			
Immobility			
	T5–T7	0.73 (0.25–2.18)	2.39 (0.85–6.72)
	T8–T10	1.90 (0.61–5.90)	1.45 (0.48–4.40)
	T11-L2	1.13 (0.41–3.14)	0.76 (0.28–2.10)

*Table entries are for risk of a positive osteopathic palpatory finding in subjects with type 2 diabetes mellitus vs. subjects without type 2 diabetes mellitus, adjusted for age, sex, hypertension, and clinical depression.

†P = .003.

First, subgroups were defined according to the presence or absence of comorbid hypertension because it is a risk factor for development of diabetic nephropathy. In subjects with type 2 diabetes mellitus and hypertension, there were significant associations with tissue changes at T11-L2 bilaterally in the logistic regression models that adjusted for age, sex, and clinical depression (OR, 27.38; 95% CI, 1.75–428; P = .02 for the left side and OR, 24.00; 95% CI, 1.51–382; P = .02 for the right side). In subjects with type 2 diabetes mellitus without hypertension, no significant associations with tissue changes at T11-L2 were observed.

Second, among subjects with type 2 diabetes mellitus with hypertension, another stratum of subgroups was created according to the duration of type 2 diabetes mellitus since its diagnosis. The duration of type 2 diabetes mellitus was defined as short (≤5 years) or long (>5 years) based on a median split. There was a strong diabetes mellitus duration effect for tissue changes at T11-L2 bilaterally (OR, 12.00; 95% CI, 1.02–141; P = .05 for short duration vs. OR, 32.00; 95% CI, 2.29–448; P = .01 for long duration on the left side; and OR, 17.33; 95% CI, 1.39–217; P = .03 for short duration vs. OR, 32.00; 95% CI, 2.29–448; P = .01 for long duration on the right side). These duration subgroup analyses are reported as crude ORs as adjustment for confounding variables was not feasible because of the relatively small number of subjects in these subgroup analyses.

## Discussion

Tissue changes, as manifested by doughy, ropy, thickened, or fibrotic interstitial tissue, at T11-L2 was the strongest and most consistent osteopathic palpatory finding associated with type 2 diabetes mellitus in this study. There are several potential explanations for this finding. First, it may reflect other phenomena in the pathogenesis or progression of type 2 diabetes mellitus, such as diabetic nephropathy. The latter would be expected to contribute to reflex viscerosomatic changes at the T11-L2 segmental level, albeit bilaterally [4]. Epidemiologic studies have refuted the notion that renal prognosis is benign in type 2 diabetes mellitus [9]. Most studies that assessed the presence of diabetic nephropathy in patients with type 2 diabetes mellitus found that at least two-thirds of patients were affected [10]. In fact, some patients may manifest with diabetic nephropathy several years prior to a diagnosis of diabetes mellitus. Although the clinical features of 15 such patients have been summarized [11], the incidence rate and natural course of this phenomenon remains unclear. Also, in our study, the significant associations with tissue changes at T11-L2 bilaterally in subjects with type 2 diabetes mellitus and hypertension may reflect an augmented viscerosomatic response to two underlying diseases that promote nephropathy.

A second possible explanation is that residual, uncontrolled confounding may have contributed to a spurious association between type 2 diabetes mellitus and osteopathic palpatory findings at the T11-L2 spinal segmental level. For example, if other diseases involving the T11-L2 spinal segmental level were more often found in subjects with type 2 diabetes mellitus than in subjects without type 2 diabetes mellitus, then these other diseases may have spuriously inflated the observed ORs. Theoretically, the anatomic structures most likely to be associated with osteopathic palpatory findings at the T11-L2 spinal segmental level include the adrenal medulla, large intestine, appendix, kidney, ureter, urinary bladder, prostate, and uterus [4]. Subgroup analyses or multivariate modeling would help address the issue of confounding; however, such analyses were limited by sample size constraints.

Finally, it is possible the observed association between type 2 diabetes mellitus and tissue changes at T11-L2 on the right side may have simply occurred by chance and may not be clinically related to type 2 diabetes mellitus at all. This appears unlikely because of the strength and consistency of the finding across analyses and because the subgroup analyses of subjects with type 2 diabetes mellitus and hypertension not only corroborated the significant association with tissue changes at T11-L2, but also demonstrated that the association was much stronger and occurred bilaterally. Additionally, among subjects with type 2 diabetes mellitus and hypertension, a strong diabetes mellitus duration effect was observed, thereby suggesting a temporal relationship between type 2 diabetes mellitus and subsequent tissue changes at T11-L2 bilaterally.

There are several limitations of this study that should be mentioned. First, this was a case-control study with a relatively small number of subjects. The inability to adequately perform more subgroup analyses or to control for additional confounders because of small sample size has already been noted. The assessment of type 2 diabetes mellitus status and examinations for osteopathic palpatory findings were performed in a cross-sectional manner. Although we hypothesize that osteopathic palpatory findings such as tissue changes at T11-L2 may be a manifestation of viscerosomatic reflexes associated with type 2 diabetes mellitus, a temporal relationship cannot definitively be established. One could argue that such osteopathic palpatory findings preceded the development of type 2 diabetes mellitus and, much like a trigger point, may have initiated somatovisceral reflexes [12]. Nevertheless, with respect to temporality, the observation of a strong diabetes mellitus duration effect for tissue changes at T11-L2 bilaterally, in conjunction with hypertension, provides a rationale to suggest the existence of a viscerosomatic reflex.

Second, the osteopathic palpatory examinations were performed by predoctoral osteopathic manipulative medicine fellows. Although these fellows elected to take an additional year of training in osteopathic manipulation during their medical curriculum and received additional study-specific training, they likely did not have the same level of proficiency in performing osteopathic palpatory examinations as more seasoned clinicians. Osteopathic students have reported more palpatory findings than physicians, presumably because more experienced examiners filter out insignificant findings [13]. A study of palpatory diagnosis found somewhat greater agreement between two osteopathic physicians than between the osteopathic physicians and a predoctoral osteopathic manipulative medicine fellow [14].

Finally, one might argue that the reliability of osteopathic palpatory findings reported in this study was not sufficiently high to ensure validity. Our initial intent was to have three examiners for each subject to allow for a majority decision on each of the 30 osteopathic palpatory findings; however, this was not logistically feasible at the time that the study was implemented. Using kappa as a frame of reference [15], many osteopathic palpatory findings in this study would be classified as having fair or poor interexaminer reliability. Four findings, all of which involved the left side, were associated with a negative kappa. Of these, the poorest level of agreement involved tissue changes at T11-L2 on the left side. Such poor reliability for this particular osteopathic palpatory finding may help explain why only unilateral (i.e., right-sided) statistically significant findings were initially observed for tissue changes at the T11-L2 spinal segmental level. Nevertheless, at the aggregate level, interexaminer reliability in this study (kappa, 0.35) was comparable to that reported for other commonly used diagnostic tests, such as exercise electrocardiograms to identify ST-T responses (kappa, 0.30) and peripheral blood films to diagnose iron-deficiency anemia (kappa, 0.39) [16]. To replicate the study findings and overcome the limitations described above, larger prospective studies with more experienced osteopathic examiners are needed.

## Conclusion

The only consistent finding in this study was an association between type 2 diabetes mellitus and tissue changes at T11-L2 on the right side. Potential explanations for this finding include reflex viscerosomatic changes directly related to the progression of type 2 diabetes mellitus, a spurious association attributable to confounding visceral diseases, or a chance observation unrelated to type 2 diabetes mellitus. Larger prospective studies are needed to better study osteopathic palpatory findings in type 2 diabetes mellitus.

## Competing interests

JCL is Editor-in-Chief of *Osteopathic Medicine and Primary Care*. STS is a member of the Editorial Board of *Osteopathic Medicine and Primary Care*. Neither was involved in the review of this manuscript or in the editorial decision with respect to its suitability for publication.

## Authors' contributions

JCL proposed the project, acquired funding, participated in the literature review, study design development, and data analysis, and was primary author of the manuscript. KGF participated in the literature review, research protocol implementation, data collection, and manuscript review. STS, RGG, and ACC participated in research protocol development and implementation and in manuscript review. All authors read and approved the manuscript.
